# Excitation and detection of coherent nanoscale spin waves via extreme ultraviolet transient gratings

**DOI:** 10.1126/sciadv.adp6015

**Published:** 2024-09-06

**Authors:** Peter R. Miedaner, Nadia Berndt, Jude Deschamps, Sergei Urazhdin, Nupur Khatu, Danny Fainozzi, Marta Brioschi, Pietro Carrara, Riccardo Cucini, Giorgio Rossi, Steffen Wittrock, Dmitriy Ksenzov, Riccardo Mincigrucci, Filippo Bencivenga, Laura Foglia, Ettore Paltanin, Stefano Bonetti, Dieter Engel, Daniel Schick, Christian Gutt, Riccardo Comin, Keith A. Nelson, Alexei A. Maznev

**Affiliations:** ^1^ Department of Chemistry, Massachusetts Institute of Technology, Cambridge, MA, USA.; ^2^ Department of Physics, Emory University, Atlanta, GA, USA.; ^3^ Elettra Sincrotrone Trieste, Basovizza, Italy.; ^4^ Department of Molecular Sciences and Nanosystems, Ca’Foscari University of Venice, Venice, Italy.; ^5^ European XFEL, Holzkoppel 4, 22869 Schenefeld, Germany.; ^6^ Dipartimento di Fisica, Università degli Studi di Milano, Milano, Italy.; ^7^ CNR-Istituto Officina dei Materiali, Trieste, Italy.; ^8^ Helmholtz-Zentrum Berlin Für Materialien und Energie GmbH, Hahn-Meitner-Platz 1, 14109 Berlin, Germany.; ^9^ Max Born Institute for Nonlinear Optics and Short Pulse Spectroscopy, Max-Born-Str. 2A, 12489 Berlin, Germany.; ^10^ Department Physik, Universität Siegen, Siegen, Germany.; ^11^ Department of Physics, Università degli Studi di Trieste, 34127 Trieste, Italy.; ^12^ Department of Physics, Stockholm University, Stockholm, Sweden.; ^13^ Department of Physics, Massachusetts Institute of Technology, Cambridge, MA, USA.

## Abstract

The advent of free electron lasers has opened the opportunity to explore interactions between extreme ultraviolet (EUV) photons and collective excitations in solids. While EUV transient grating spectroscopy, a noncollinear four-wave mixing technique, has already been applied to probe coherent phonons, the potential of EUV radiation for studying nanoscale spin waves has not been harnessed. Here we report EUV transient grating experiments with coherent magnons in Fe/Gd ferrimagnetic multilayers. Magnons with tens of nanometers wavelengths are excited by a pair of femtosecond EUV pulses and detected via diffraction of a probe pulse tuned to an absorption edge of Gd. The results unlock the potential of nonlinear EUV spectroscopy for studying magnons and provide a tool for exploring spin waves in a wave vector range not accessible by established inelastic scattering techniques.

## Teaser

Extreme ultraviolet four-wave mixing mediated by coherent spin waves offers a spectroscopic tool for magnonics.

## INTRODUCTION

The interaction of electromagnetic radiation with collective excitations in solids such as magnons and phonons forms the basis for well-established spectroscopic techniques such as Brillouin light scattering (BLS) and inelastic x-ray scattering. However, a large wavelength gap between BLS and x-ray scattering has remained largely unexplored, primarily due to a lack of high-resolution extreme ultraviolet (EUV) spectrometers. The advent of free electron lasers (FELs) has enabled the investigation of nonlinear interactions of short-wavelength (EUV and x-ray) femtosecond pulses in solids (*1*–*5*). In particular, EUV transient grating (TG) spectroscopy has been demonstrated, and a dedicated setup has been constructed at the FERMI FEL in Trieste, Italy (*6*, *7*). In this noncollinear four-wave mixing technique, spatially periodic material excitations generated by two crossed EUV pump pulses act as a transient diffraction grating that scatters a time-delayed EUV probe pulse. While initial EUV TG studies involved periodic temperature modulations and coherent phonons (*7*–*9*), the technique was recently extended to study transient gratings of magnetization (TMGs) by using a probe wavelength resonant with an absorption edge of a magnetic element (*10*, *11*). This development opens the possibility for EUV four-wave mixing mediated by magnons, the fundamental collective excitations of long-range magnetic ordering, in which a pair of femtosecond EUV pump pulses excites coherent spin waves whose dynamics are monitored via resonant scattering of an EUV probe pulse. This would enable excitation and detection of coherent magnons with nanoscale wavelengths not accessible to existing inelastic scattering techniques. Furthermore, unlike linear scattering spectroscopies probing thermal magnon population, TMG spectroscopy would involve the generation and detection of coherent magnons. While optical femtosecond excitation of coherent magnons with zero in-plane wave vector has been well studied (*12*), and the excitation of finite wave vector coherent magnons by crossing two optical pulses has recently been demonstrated (*13*), the use of EUV radiation would allow access to much higher magnon wave vectors, which are essential for the development of high-speed and nanoscale magnonic devices (*14*, *15*).

In this work, we describe EUV TMG experiments involving coherent spin waves with nanoscale wavelengths in rare earth-transition metal (RE-TM) ferrimagnetic multilayers. We vary the pump wavelength to access distinct excitation wave vectors while fixing the probe wavelength at the absorption edge of the RE element to ensure resonant scattering from the magnetization grating. EUV TMG data combined with optical pump-probe measurements of zero wave vector magnons are used to construct spin wave dispersions extending up to 0.12 nm^−1^. Our results demonstrate the potential of EUV radiation for studying magnons and introduce EUV TMG as a tool for high wave vector coherent magnon spectroscopy.

## RESULTS

**Fig. 1.**
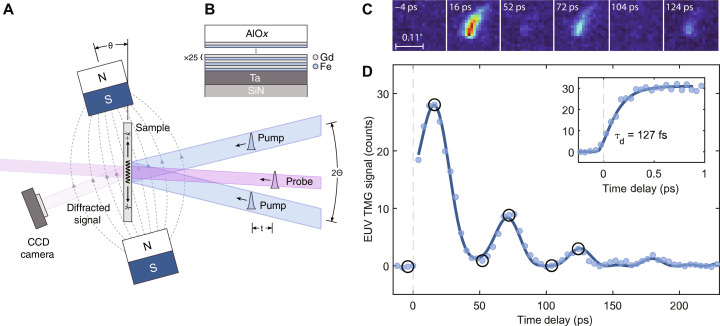
Experimental overview. (**A**) Experimental setup. Two crossed EUV pulses (blue) generate counter-propagating spin waves with wave vectors ±*k*. A time delayed EUV probe pulse (pink) is diffracted by the spatially periodic modulation of the magnetization producing a signal on the CCD camera. An external DC magnetic field is applied to tilt the magnetization direction. (**B**) A schematic of the Fe/Gd multilayer sample structure. (**C**) CCD images of the diffracted probe intensity at different time delays for the CMA sample at a magnetic field angle of θ = 15° and a grating period of Λ = 52.5 nm. The panels show the same small region of the CCD, with the scale bar corresponding to 270 μm or 0.11° in terms of scattering angle. The horizontal axis lies in the scattering plane [i.e., the plane of the drawing in (A)]. (**D**) Integrated signal versus pump-probe delay. Circled points correspond to the images shown in (C). The solid curve is a fit by Eq. 1. The initial dynamics measured with a 50-fs time step are shown in the inset, with the solid curve being a fit by Eq. 3.

TMG measurements were performed at the EIS-TIMER beamline of the FERMI FEL (*6*, *7*). The experimental setup is shown in Fig. 1A, and additional details are provided in Materials and Methods. Two time-coincident EUV pump pulses with wavelength λ_ex_ were crossed at an angle 2Θ = 27.6°, generating a sinusoidal intensity profile with a period of $\Lambda =\frac{\lambda_{\text{ex}}}{2\text{sin}\Theta }.$ The pump wavelength was varied from 8.34 to 41.7 nm, producing a discrete set of TMG periods Λ = 17.5, 52.5, 69.9, and 87.4 nm.

The dynamics of the transient spatially periodic magnetization pattern produced by the pump pulses were probed via diffraction of a time-delayed probe pulse with wavelength λ_pr_ = 8.34 nm resonant with the N_4,5_-edge of Gd, which has been shown to yield large magnetooptical coefficients (*16*, *17*). Thus, the observed magnetic signal was selectively sensitive to the Gd spin sublattice. The samples were placed in an external magnetic field of 250 mT, coplanar with the scattering plane and applied at a variable angle θ with respect to the sample surface.

The investigated samples were two Fe/Gd ferrimagnetic multilayers whose structure is shown in Fig. 1B. The magnetization in both samples was Gd dominated. In one sample, the in-plane anisotropy due to the demagnetization field was compensated by the interfacial perpendicular anisotropy contribution. We denote this sample as CMA referring to its compensated magnetic anisotropy (not to be confused with compensated magnetization). The other sample with higher Fe content was closer to the magnetic compensation point and exhibited a smaller magnetization (see Supplementary Text and figs. S1 and S2). Because of the smaller demagnetizing field, it exhibited perpendicular magnetic anisotropy, and we thus refer to this sample as PMA.

Figure 1 (C and D) shows the data collected from the CMA sample at Λ = 52.5 nm. The raw data, i.e., charge-coupled device (CCD) images of the diffracted signal, are shown in Fig. 1C, while Fig. 1D shows the integrated signal intensity versus the pump-probe time delay. At negative delays, the signal is absent. The absorption of the pump EUV pulses leads to a local electronic temperature increase at the TG maxima (i.e., the maxima of the pump interference pattern), which results in partial demagnetization. The grating formed by this periodic magnetization modulation scatters the time-delayed probe at an angle corresponding to a wave vector transfer of magnitude $k=\frac{2\pi }{\Lambda }$ . The scattering angles for the different TMG periods are listed in Table 1. The diffracted beam yields a small spot on the CCD whose size reflects the footprint of the probe beam. (Any wave vector spread is negligible compared to the latter). The rise time of the signal in the inset indicates that the demagnetization occurs in τ_d_ ∼ 130 fs (see Materials and Methods for details). This time is notably shorter than the demagnetization time of the Gd sublattice in Gd-transition metal ferrimagnets reported in prior experiments with optical excitation (*18*, *19*). Whether the observed fast demagnetization of Gd is specific to EUV excitation and might be related, for example, to the direct excitation of magnetic 4*f* electrons in Gd, is an open question calling for further investigation. After ~1 ps, both the demagnetization and electron-phonon relaxation are completed, and thermal equilibrium among electrons, spins, and the lattice is locally established. We estimated that the temperature rise at the TG maxima at this point is ∼50 K (see Supplementary Text). This temperature rise also causes a change in the magnetic anisotropy, resulting in a deviation of the effective field **H**_eff_ from the initial magnetization direction. This initiates precession of the magnetization vector about the new **H**_eff_ direction (*12*, *20*). Since in our case precession is driven in a spatially periodic pattern, it launches counter-propagating coherent spin waves at the TG wave vector *k*, causing the diffracted signal to oscillate at a frequency of 18 GHz as is clearly evident in Fig. 1D. Meanwhile, thermal transport washes out the magnetization grating associated with the temperature profile, which results in the slowly decaying component of the signal. Since the diffracted signal intensity is quadratic with respect to the amplitude of the magnetization grating (*10*), its time dependence *S*(*t*), following the initial demagnetization, can be described by

$$S\left(t>0\right)={\left[a_{0}e^{-\frac{t}{\tau }}+a_{1}e^{-\alpha t}\text{sin}\left(2\pi \mathrm{\nu t}-\varphi \right)\right]}^{2}$$ (1)

where τ is the thermal relaxation time, ν and α are the wave vector–dependent spin wave frequency and damping rate, respectively, and φ is a phase factor. The first term describes the decay of the magnetization grating via thermal transport, while the second describes the spin wave oscillations. As can be seen from Fig. 1D, Eq. 1 provides a reasonable fit to the data. Note that it is the presence of the first term that allows one to see oscillations at the spin wave frequency ν; on its own, the standing wave formed by counter-propagating spin waves would yield a signal oscillating at 2ν, which is indeed visible in the tail of the waveform in the figure.

**Table 1.** EUV TMG experimental configurations. FWHM, full width at half maximum.

Configuration	1	2	3	4
Pump wavelength λ_ex_ (nm)	8.34	25.02	33.34	41.7
Grating period Λ (nm)	17.5	52.5	69.9	87.4
Probe scattering angle to sample normal (°)	23.4	4.5	2.2	0.9
FWHM pump spot size (μm)	180	300	300	300
Pump energy at the sample (μJ)	0.04	0.34–0.38	0.21–0.36	0.44–0.72

**Fig. 2.**
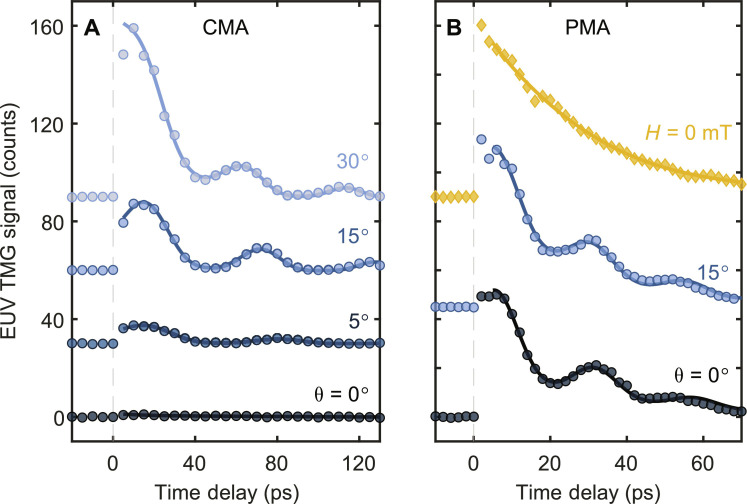
Magnetic field dependence. (**A**) TMG responses versus the applied magnetic field angle θ for the CMA sample. (**B**) TMG responses for the PMA sample measured at two representative applied field angles and without the applied field (yellow dots). Data are displaced vertically for clarity. Solid curves are fits by Eq. 1.

The TMG signal is polarized orthogonally with respect to the incident probe beam (*10*) and can be separated from the nonmagnetic TG responses (*11*). However, our setup did not include a polarizing mirror after the sample (*11*); therefore, in principle, our signal could contain contributions of nonmagnetic origin such as electronic and thermoelastic responses (*6*, *10*). However, the dependence of the signal from the CMA sample on the magnetic field angle shown in Fig. 2A indicates that the contribution of nonmagnetic responses to the signal is negligible. When the field is in the plane of the sample (θ=0°), no TMG signal is observed, as the magnetization is almost orthogonal to the probe propagation direction. As the field is rotated out of plane, both the longitudinal (demagnetization) and transverse (coherent spin precession) responses increase, providing unambiguous evidence that the observed signal is of magnetic origin. The photon flux in the probe beam was much smaller than in the previous TMG experiment (*10*), where weak electronic and thermoelastic responses were observed alongside the magnetic response on a CoGd alloy.

Furthermore, we did not detect a TMG signal from a 15-nm-thick film of pure Gd, which is not magnetic at 300 K. These observations are consistent with previous results (*11*) where the response of a FeGd alloy sample probed at the N_4,5_-edge of Gd was shown to be purely magnetic. This should also be true for the PMA sample as the electronic and thermoelastic responses are not sensitive to small changes in composition. In contrast to the CMA sample, the signal from the PMA sample does not appreciably change with the change of the applied field angle between 0° and 15°, as shown in Fig. 2B. However, when the magnetic field is removed, the oscillations vanish, again indicating clearly that the transverse dynamics are of magnetic origin. The presence of the longitudinal response at zero external field is due to the remanent out-of-plane magnetization of the sample.

**Fig. 3.**
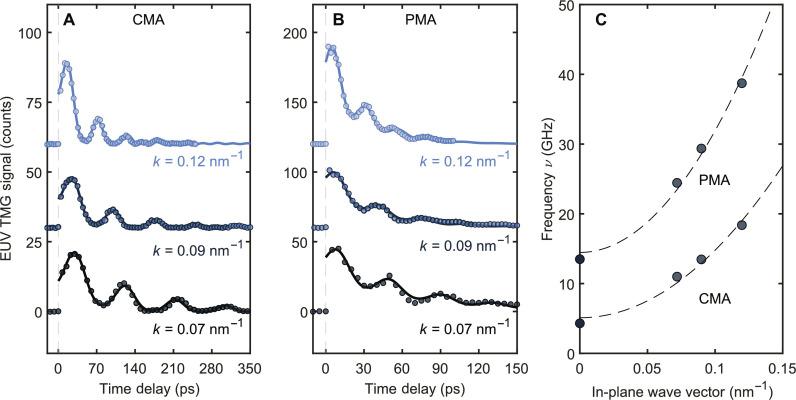
Spin wave dispersion. (**A** and **B**) Time-domain data for CMA (A) and PMA (B) samples at the labeled wave vectors. Solid curves represent fits by Eq. 1. (**C**) Spin wave dispersions, ν(*k*), for the two samples. Dashed curves represent fits by Eq. 2

To determine the spin wave dispersions of both samples, three different values of λ_ex_ were used, producing TMG periods of 52.5, 69.9, and 87.4 nm. The time-dependent responses are shown in Fig. 3 (A and B), while Fig. 3C shows the values of ν(*k*) obtained by fitting Eq.1 to the time-domain waveforms. Measurements were performed at θ = 15° for the CMA sample to maximize the transverse response, while for the PMA sample, the most complete set of measurements was performed at θ = 0°. In addition to EUV measurements, optical pump-probe measurements with Faraday rotation detection (*21*, *22*) were performed under identical magnetic fields to determine zero wave vector spin wave frequencies (see Supplementary Text and fig. S3).

The dispersion data in Fig. 3C were fitted by the ferromagnetic dispersion relation

$$\nu \left(k\right)=\Delta +Dk^{2}$$ (2)

where Δ and *D* are the zone-center magnon gap and magnon stiffness, respectively. For a ferrimagnet, Δ and *D* are given by equation 119 of (*23*). The quadratic behavior at high *k* is characteristic for spin waves dominated by the exchange interaction (*24*). A dipole contribution neglected in Eq. 2 may explain small deviations from the fit at low wave vectors. The value of Δ is about three times larger for the PMA sample, which is closer to the angular momentum compensation point. A similar increase of the ferromagnetic resonance frequency (FMR) at compositions approaching the magnetic compensation point was reported for Gd-TM alloys (*22*, *25*).

The magnon stiffness determined from the dispersion is *D* = 1000 GHz nm^2^ for the CMA sample and 1700 GHz nm^2^ for the PMA sample. (For reference, the spin wave stiffness in pure Fe is ∼600 GHz nm^2^ (*26*).) While FMR in RE-TM alloys has been studied extensively (*21*, *22*, *25*, *27*), there are no literature data on the spin wave dispersion in the exchange-dominated region in these materials, as the measurements are normally performed on thin films which are unsuitable for inelastic neutron scattering. The higher spin wave stiffness of the PMA sample confirms the longstanding prediction that the spin wave stiffness diverges at the angular momentum compensation point (*23*).

The spin wave group velocity can be directly extracted from the dispersion (*v_g_* = 2*Dk*). At the largest experimental wave vector, 0.12 nm^−1^, we estimate *v_g_*= 1.5 km/s for the CMA sample and *v_g_*= 2.6 km/s for the PMA sample. Despite only slight changes in the material composition, the two samples exhibit substantial differences in spin wave propagation speeds, suggesting an approach to efficiently control spin wave propagation in magnonic applications.

**Fig. 4.**
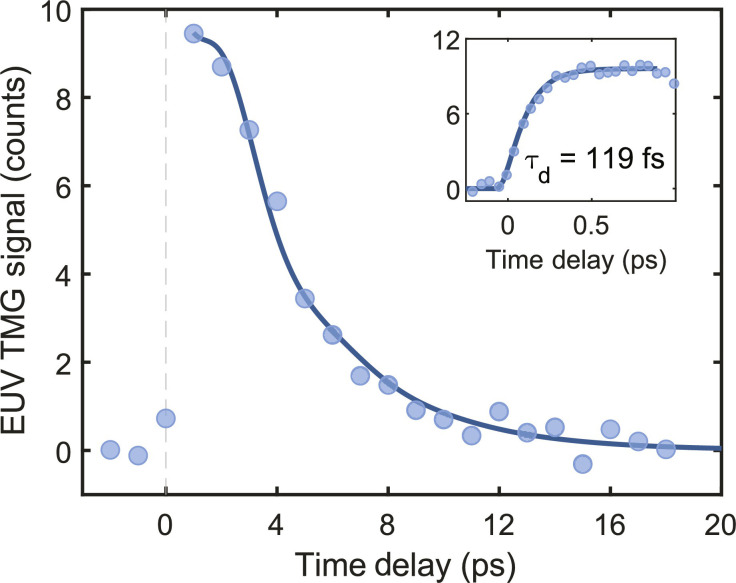
Short-period TMG. Diffracted signal from the PMA sample with a TMG period of 17.5 nm at θ = 15°. The solid curve is a fit by Eq. 1 with the frequency set to ν = 230 GHz, resulting in an overdamped oscillation on top of an exponential decay. A fine time step scan is shown in the inset, where the solid curve is a fit by Eq. 3.

We also conducted measurements at a 17.5 nm TMG period (λ_ex_ = λ_pr_ = 8.34 nm) but observed no distinct magnon oscillations, as seen in Fig. 4. In these measurements, the signal-to-noise ratio was smaller than at larger TMG periods, as the pump fluence was lower, and parasitic scattering from the pump beams could not be filtered out in this degenerate pump-probe configuration. However, the response clearly deviates from an exponential decay, suggesting the presence of overdamped spin waves. Equation 1 with the magnon frequency set to a value of 230 GHz obtained by extrapolating the trend observed in Fig. 3 to *k* = 0.36 nm^−1^ yields a reasonable fit to the signal waveform in Fig. 4 at α = 530 GHz. However, measurements at intermediate wave vectors would be needed to confirm such an interpretation of the data. Nevertheless, the transient diffraction signal in Fig. 4 clearly indicates the formation of a periodic magnetic texture at this short period. The corresponding width of demagnetized regions (i.e., half-pitch of the TMG) is less than 10 nm. The ultrafast demagnetization time (see inset in Fig. 4) was found to be τ_d_ ∼ 110 fs, similar to that observed at longer TMG periods (see fig. S4). This indicates that spin diffusion, suggested previously as a mechanism for ultrafast demagnetization in ferromagnetic metals (*28*, *29*), is unlikely to contribute to the formation of the transient magnetization gratings in our experiment: Otherwise, the dynamics would have depended on the TMG period. The thermal relaxation time of ∼7.5 ps is much shorter than at longer TMG periods, roughly in agreement with the expected quadratic dependence of the thermal decay time on Λ (see supplementary text and fig. S5) (*6*).

## DISCUSSION

EUV TMG-based four-wave mixing enables a magnon spectroscopy capable of filling in the wave vector gap between Brillouin scattering covering the wave vector range of up to 0.03 nm^−1^ (*30*–*32*) and inelastic neutron scattering covering large wave vectors above 0.5 nm^−1^ (*33*). The former is generally limited to long wavelength dipole spin waves characterized by smaller group velocities, which limits its usefulness for magnonic applications, while the latter is restricted to bulk samples. As a time-domain technique, EUV TMG is not limited by the spectrometer resolution. The practical resolution limit is set by the scanning range of the delay line: For example, the 1000-ps delay range available at FERMI corresponds to a frequency resolution of about 1 GHz or 4 μeV. For comparison, the state-of-the-art resolution of inelastic neutron scattering spectroscopy of magnons is about 1 meV (*34*), which is not nearly sufficient to resolve the magnon frequencies measured in the present study. Resonant inelastic soft x-ray scattering has recently been adopted for studying large wave vector magnons and is suitable for studying thin-film samples but has so far only achieved a resolution in the tens of milli–electron volt range (*35*).

In the context of magnonics (*15*), recent efforts were directed toward coherent magnons with wavelengths under 100 nm. Existing methods involve fabricating nanostructures on the sample to achieve phase matching between the driving long-wavelength microwaves and short-wavelength spin waves (*36*, *37*). TMG spectroscopy offers a versatile and noninvasive approach not constrained by the limitations of nanofabrication. Furthermore, the ability to impulsively excite large amplitude spin waves may open doors to studies of nonlinear phenomena such as spin wave mixing (*38*–*40*) at short wavelengths. In addition, the EUV TG technique can be used to study the interaction of short-wavelength coherent magnons with impurities, domain walls, and nanostructures. The high damping rates in the samples used in this study resulted in small magnon propagation lengths on the order of a couple of wavelengths. However, this technique can be used for materials with long magnon mean free paths such as yttrium iron garnet (*36*). Measurements of magnon propagation on such samples could be further facilitated by a spatial separation of the excitation and probe spots. While the results reported here were obtained in transmission geometry that requires the samples to be in the form of ultrathin membranes, EUV TG spectroscopy of surface phonons (*8*) and transient magnetic polarization gratings (*41*) has already been demonstrated in reflection geometry. Although the demonstrated method is now limited by the need of a large FEL facility, it is foreseeable that this technique could be replicated on a tabletop with high-harmonic generation methods (*42*).

In addition to spin wave spectroscopy, we have also demonstrated the ability to use the TMG technique to create magnetic textures on the sub-10-nm scale. Further investigations of magnetic dynamics at this scale will push the limits of ultrafast nanoscale magnetism. In particular, such studies may reveal the interplay of spin and thermal transport in transient magnetic textures, which would both inform theoretical models and improve our understanding of the lifetimes of magnetic storage devices. Although these gratings are transient, stable magnetic textures have been generated using the TMG method (*43*, *44*), which may provide a noninvasive approach to dense magnetic data storage.

In summary, we have demonstrated resonant magnon-mediated EUV four-wave mixing that unlocks the potential of EUV radiation for studying spin waves. Coherent magnon spectroscopy based on the EUV TMG approach overcomes the limitations of existing techniques and provides the means to broaden our understanding of ultrafast magnetic dynamics at the nanoscale and to facilitate research toward high-speed magnonic devices.

## MATERIALS AND METHODS

### Experimental design

Details of the experimental TG setup at FERMI can be found in (*45*). The seeded FEL outputs nearly transform-limited EUV pulses of 50 fs in duration and can operate in a dual-wavelength regime which permits using different wavelengths for pumping and probing (*45*, *46*). The parameters of the pump pulses for the experimental configurations used in this study are summarized in Table 1; the polarization of the pump pulses was circular, which is preferred for stable operation of the FEL. The probe energy at the sample was ∼0.01 μJ with a full width at half maximum spot size of ~120 μm, and its polarization was linear vertical (i.e., orthogonal to the plane of the drawing in Fig. 1A). The probe incident angle was Θ*_pr_* = 4.6° defined from the sample normal, meaning that the signal was predominantly sensitive to the out-of-plane magnetization component. The two pump beams, the probe, and the magnetic field were all coplanar normal to the sample surface. The sample was thin enough to ensure diffraction in the “thin grating” regime; hence, the scattering geometry did not have to satisfy the Bragg condition. Images of the diffracted probe were collected by a CCD camera, averaging 2000 shots of the FEL, which operates at a repetition rate of 50 Hz. The distance between the camera and the sample was 140 mm. For each TMG period, the CCD was centered at the expected scattering angle. The measurements were conducted at room temperature in ultrahigh vacuum (10^−7^ mbar).

When varying the pump wavelength, it is important to consider the dependence of ν on the pump fluence observed previously in optical pump-probe measurements on ferrimagnetic TM-RE alloys (*22*). If this effect is pronounced, then in experiments with different pump wavelengths one should match the absorbed energy density rather than fluence, since the absorption length varies with λ_ex_. In our EUV measurements, we mitigated this issue by adjusting the pump fluence to maintain the magnitude of the initial fast rise of the signal approximately equal for different values of λ_ex_. We have also performed fluence-dependent measurements, which did not show any substantial dependence of the spin wave frequency on the pump fluence at EUV and optical wavelengths (see fig. S6). We conclude that the fluences were too low to affect the spin wave frequencies via sample heating. At higher fluences, in both EUV and optical measurements, the spin wave oscillations become less pronounced compared to the longitudinal response and eventually disappear. The nature of this effect also reported in (*21*) is not entirely clear and warrants further investigation.

### Sample preparation

The Fe/Gd multilayers had the structure Ta (2)[Fe(*x*)Gd(0.8-*x*)]×25AlO*x*(2.5), where the numbers in parentheses are thicknesses in nanometers. The Fe thickness was 0.36 nm for the CMA sample and 0.38 nm for the PMA sample. As the Fe and Gd thicknesses are under 1 nm, the interlayer diffusion prevents the formation of well-defined layers. While we use the term “multilayer” in this manuscript, they are more accurately described as concentration-modulated alloys. The multilayers were deposited on SiN(50) membranes by sequential sputtering. The CMA sample exhibited zero in-plane and out-of-plane coercive fields and a magnetization of *M* ∼ 1.8 × 10^5^ A/m. The PMA sample exhibited an in-plane coercive field of *H*_IP_ ~ 300 mT, an out-of-plane coercive field of *H*_OOP_ ~1.5 mT, and a magnetization of *M*∼10^5^ A/m (see hysteresis curves in fig. S1.)

### Data processing and analysis

The signal was integrated within an elliptical region of interest set around the signal spot on the CCD. A region on the CCD where no signal was present was used as a reference for background subtraction. In addition, we subtracted a background obtained by averaging the data points collected at negative time delays. The resulting data were normalized by the square of the pump intensity to reduce the noise arising from FEL fluctuations. Longer timescale dynamics were measured with 5-ps steps until the signal had decayed and fitted by Eq. 1. Subpicosecond dynamics (i.e*.*, ultrafast demagnetization) were measured using 50-fs steps until the signal plateaued and were fit to an exponential function to extract the demagnetization time τ_d_ in a way consistent with literature (*18*, *19*)

$$S\left(t\right)={\left[\mathrm{aH}\left(t-t_{0}\right)\left(1-e^{-\frac{\left(t-t_{0}\right)}{\tau_{d}}}\right)\right]}^{2}$$ (3)

where *a* is a scaling factor, *H*(*t*) is the Heaviside step function, and *t*_0_ is the true time zero. The equation used in (*18*, *19*) is squared here to account for the fact that the diffracted signal is quadratic with respect to the amplitude of the magnetization grating.

For the magnon dispersion measurements, we collected two to four pump-probe delay scans for each wave vector and used their average to produce the data shown in Fig. 3. The statistical error of the spin wave frequency measurements was then estimated on the basis of the analysis of individual scans (see Supplementary Text and fig. S7). Although the number of scans at each wave vector was too small to make a rigorous statistical analysis, the entire dataset provides a rough estimate of the overall error: Calculating the SD in the magnon frequency at each wave vector and averaging over the three wave vector points yields an error estimate of 0.07 GHz for the CMA sample and 0.7 GHz for the PMA sample.

## Supplementary Materials

### This PDF file includes:

Supplementary Text Figs. S1 to S7 References
