# Supplementary material for: Excitation and detection of coherent nanoscale spin waves via extreme ultraviolet transient gratings
Source: Sci Adv. 2024 Sep 6;10(36):eadp6015. doi: 10.1126/sciadv.adp6015 (PMC11378899; doi:10.1126/sciadv.adp6015)
Supplement: Supplementary file 1 — Supplementary Text Figs. S1 to S7 References [file sciadv.adp6015_sm.pdf]

Science Advances
AAAS

Supplementary Materials for

Excitation and detection of coherent nanoscale spin waves via extreme ultraviolet transient gratings

Peter R. Miedaner *et al.*

Corresponding author: Alexei A. Maznev, alexei.maznev@gmail.com

Sci. Adv. **10**, eadp6015 (2024)
DOI: 10.1126/sciadv.adp6015

This PDF file includes:

Supplementary Text
Figs. S1 to S7
References

Supplementary Text

Sample Preparation

The two samples used in the study were chosen from a set of six samples with composition Ta(2)[Fe(x)Gd(0.8 − x)]x25AlOx(2.5) grown with slightly different layer thicknesses, x = 0.36, 0.38, 0.40, 0.42, 0.44, and 0.46 nm. The multilayers were deposited on 50 nm thick SiN membranes on Si frames with a suspended area 2 mm x 2 mm by sequential sputtering from stoichiometric targets in an ultrahigh vacuum deposition chamber with the base pressure of $3 \cdot 10^{-9}$ Torr, in ultrahigh purity Ar processing gas that was additionally purified using an inert gas purifier. (Separate testing showed that non-inert background gas pressures above 10^{-6} Torr during deposition resulted in reduced magnetic moments of Fe sublattice, as documented in prior studies of sperimagnetism in FeGd alloys.) All metals were deposited by DC sputtering, and AlOx(2.5) by RF sputtering, with deposition rates between 0.2 and 0.6 A/s, which were calibrated to precision of better than 5% using a quartz crystal microbalance.

Vibrating sample magnetometry was used to obtain the magnetization of all samples plotted against Fe layer thickness in Fig. S2. One can see that at room temperature, the sample set spans the magnetic compensation point, which is closest to the x = 0.44 nm sample. Hysteresis loops for the two samples used in the experiment are shown in Fig. S1.

Optical Pump-Probe Faraday Rotation Experiment

Zero-wavevector optical measurements were performed following the standard scheme for ultrafast Faraday rotation (21, 22). The 3 mJ output of an amplified Ti:Sapphire laser with a central wavelength of 800 nm, pulse duration of 100 fs, and repetition rate of 1 kHz was split into 90% pump and 10% probe. The frequency-doubled 400 nm pump beam was incident on the sample a few degrees off the normal, with a FWHM spot size of 400 um and a fluence of 0.06 mJ/cm^2. The pump was time-delayed using an optical delay line. The 800 nm linearly polarized probe was focused with normal incidence onto the sample onto a spot of 80 μm FWHM and overlapped with the pump spot. A spatial filter and long pass filters blocked the pump light that was transmitted through the sample, and the polarization components of the transmitted probe beam were separated using a Wollaston prism. The two polarization branches were sent to a pair of photodiodes, and the difference signal was collected using a lock-in amplifier. The setup was balanced to set the difference signal to zero when no magnetic sample was present. Permanent magnets provided an external magnetic field of 250 mT rotated by the angle θ with respect to the sample plane to match the conditions used in the EUV TMG measurements. All measurements were performed at room temperature. Fig. S3 shows the Faraday rotation signals for the compensated magnetic anisotropy (CMA) and perpendicular magnetic anisotropy (PMA) samples, whose frequencies were used as the zero-wavevector points in the magnon dispersion (Fig. 3C).

Temperature Grating Estimate

The temperature rise at the transient grating maxima can be estimated by $\Delta T = 2q/c$, where q is the average absorbed energy density calculated based on the incident pump fluence and the optical properties of the constituent layers at a given EUV pump wavelength (47), and c the heat capacity of the FeGd multilayer structure per unit volume. Over all TMG periods and energies listed in Table 1, the temperature rise ranged from 36-180 K.

Thermal Relaxation Time

In a one-dimensional thermal diffusivity model, the expected thermal grating relaxation time τ is quadratic with respect to the TMG period (6).

$$\tau = \Lambda^2/4\pi^2\alpha_{\text{therm}} \tag{S1}$$

where α_{therm} is the thermal diffusivity. Figure S5 shows the measured decay times along with parabolic fits according to Eq. S1, with $\alpha_{\text{therm}} = 1.1 \times 10^{-6}$ m²/s and 1.5×10^{-6} m²/s for the CMA and PMA samples, respectively.

<u>Diffraction Angle Calculation</u>

The angle of the diffracted signal is given by the thin grating equation (48)

$$\Lambda\left(\sin(\theta_{pr}) - \sin(\theta_{sig})\right) = \lambda_{pr}$$

where Λ is the TMG period, θ_{sig} the signal diffraction angle, θ_{pr} the incident probe angle, m the diffraction order, and λ_{pr} the probe wavelength. In our setup, the sign of θ_{sig} is opposite to that of θ_{pr} and its values are listed in Table 1 of the main manuscript.

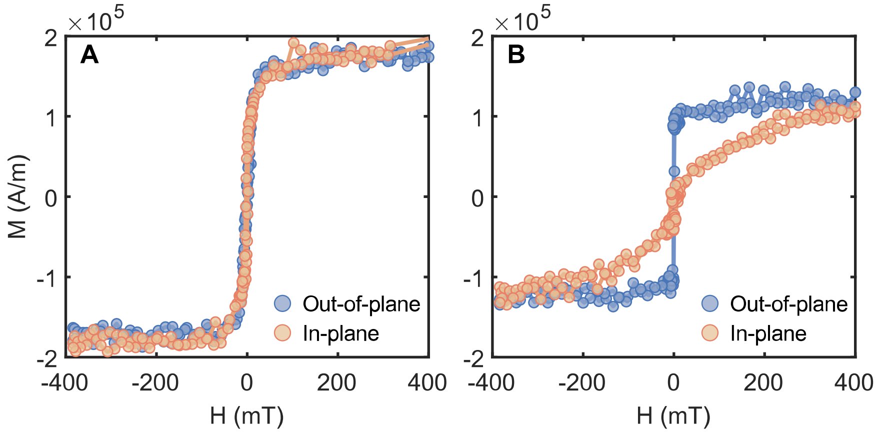

Fig. S1. Hysteresis curves.

A CMA sample with zero in-plane and out-of-plane coercive fields, and **B** PMA sample with an in-plane coercive field of ~ 300 mT and out-of-plane coercive field of ~1.5 mT.

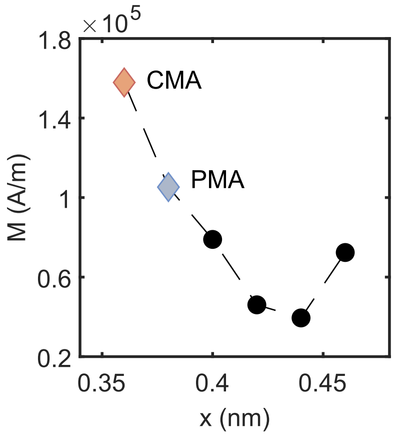

Fig. S2. Magnetization dependence of Fe layer thickness.

Diamond markers represent the two samples used in the experiment, where x = 0.36 nm is the CMA sample and x = 0.38 nm the PMA sample. The x = 0.44 nm sample is closest to the magnetic compensation point at room temperature.

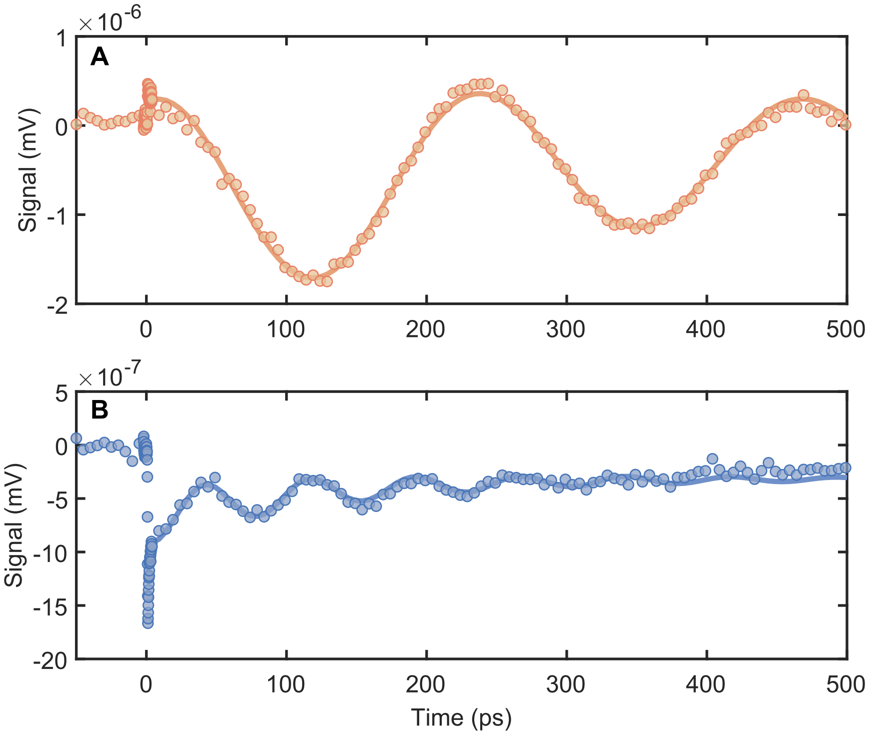

Fig. S3. Zero-wavevector Faraday rotation measurements.

A CMA sample at θ = 15° with a frequency 4.35 ± 0.02 GHz and **B** PMA sample at θ = 0° with a frequency of 13.5 ± 0.2 GHz.

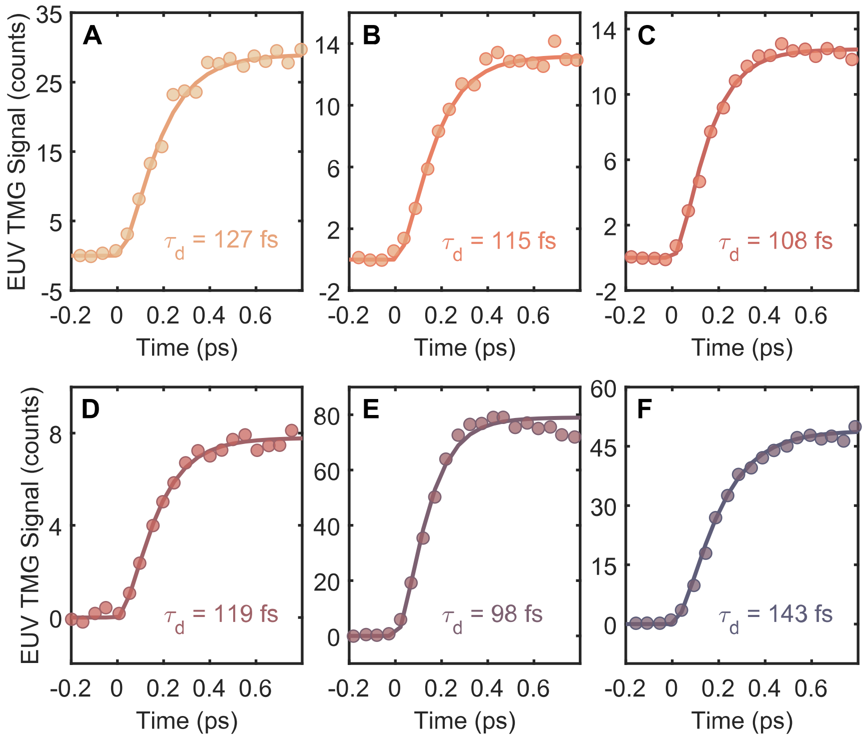

Fig. S4. Ultrafast demagnetization rise times.

TMG signal close to t = 0 measured with a 50 fs time steps **A-C** for the CMA sample with TMG periods $\Lambda = 52.5, 69.9, 87.4$ nm respectively. **D-F** PMA sample with TMG periods Λ = 17.5, 52.5, 69.9 nm respectively.

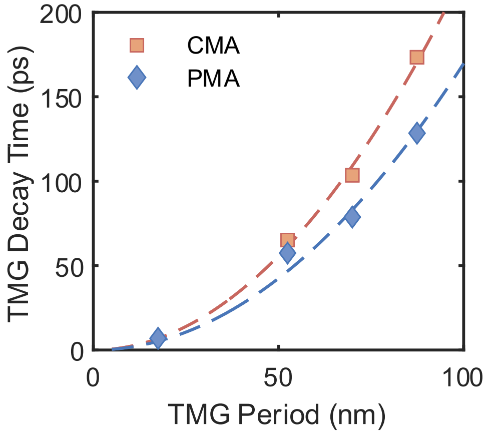

Fig. S5. TMG decay times.

Decay time vs. TMG period. Dashed lines represent parabolic fits according to Eq. S1.

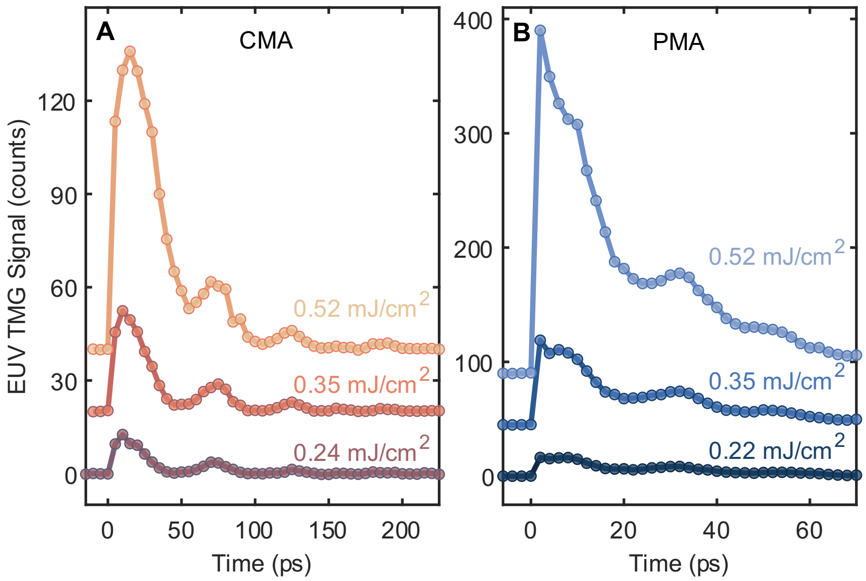

Fig. S6. Pump fluence dependence of TMG dynamics.

EUV TMG responses for Λ = 52.5 nm vs pump fluence at the sample for **A** CMA and **B** PMA samples. Plots are displaced vertically for clarity.

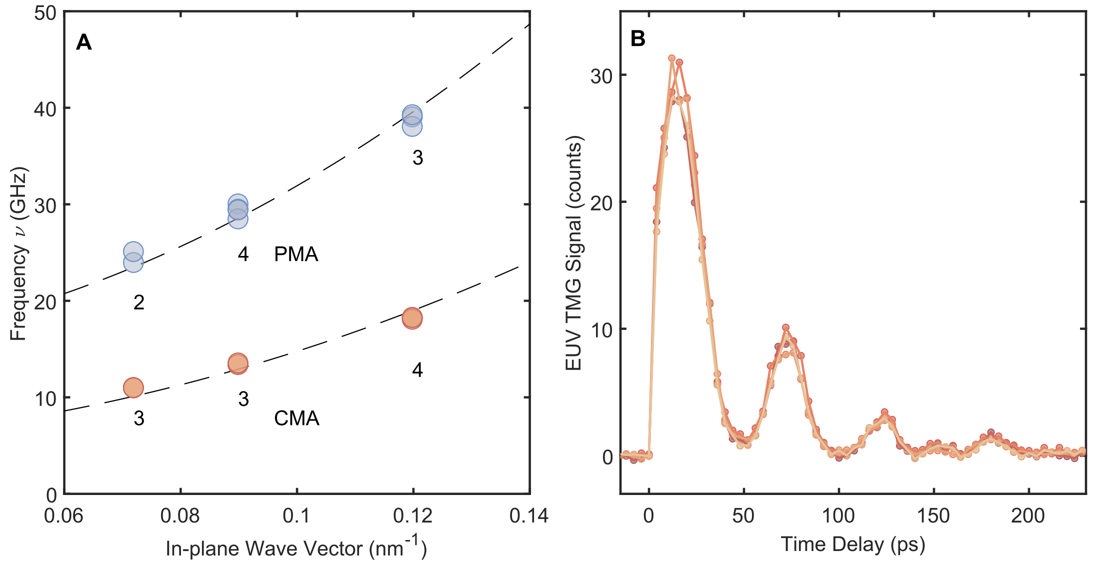

Fig. S7. Individual pump-probe delay scans for statistical error estimate.

A Magnon dispersion data obtained from individual scans. The number of scans taken at each wave vector for each sample is indicated in the figure. Note that in some cases the symbols corresponding to individual scans overlap. Dashed lines are the same as in Fig. 3C. **B** Four individual scans for CMA sample at $\Lambda = 52.5$ nm illustrating repeatability of the measurements.

REFERENCES AND NOTES

1. T. E. Glover, D. M. Fritz, M. Cammarata, T. K. Allison, S. Coh, J. M. Feldkamp, D. Zhu, Y. Feng, R. N. Coffee, M. Fuchs, S. Ghimire, J. Chen, S. Shwartz, D. A. Reis, S. E. Harris, J. B. Hastings, X-ray and optical wave mixing. *Nature* **488**, 603–608 (2012).

2. S. Shwartz, M. Fuchs, J. B. Hastings, Y. Inubushi, T. Katayama, D. A. Reis, T. Sato, K. Tono, M. Yabashi, S. Yudovich, S. E. Harris, X-ray second harmonic generation. *Phys. Rev. Lett.* **112**, 163901 (2014).

3. F. Bencivenga, R. Cucini, F. Capotondi, A. Battistoni, R. Mincigrucci, E. Giangrisostomi, A. Gessini, M. Manfredda, I. P. Nikolov, E. Pedersoli, E. Principi, C. Svetina, P. Parisse, F. Casolari, M. B. Danailov, M. Kiskinova, C. Masciovecchio, Four-wave mixing experiments with extreme ultraviolet transient gratings. *Nature* **520**, 205–208 (2015).

4. L. Foglia, F. Capotondi, R. Mincigrucci, D. Naumenko, E. Pedersoli, A. Simoncig, G. Kurdi, A. Calvi, M. Manfredda, L. Raimondi, N. Mahne, M. Zangrando, C. Masciovecchio, F. Bencivenga, First evidence of purely extreme ultraviolet four-wave mixing. *Phys. Rev. Lett.* **120**, 263901 (2018).

5. M. Chergui, M. Beye, S. Mukamel, C. Svetina, C. Masciovecchio, Progress and prospects in nonlinear extreme-ultraviolet and x-ray optics and spectroscopy. *Nat. Rev. Phys.* **5**, 578–596 (2023).

6. F. Bencivenga, R. Mincigrucci, F. Capotondi, L. Foglia, D. Naumenko, A. A. Maznev,E. Pedersoli, A. Simoncig, F. Caporaletti, V. Chiloyan, R. Cucini, F. Dallari, R. A. Duncan,T. D. Frazer, G. Gaio, A. Gessini, L. Giannessi, S. Huberman, H. Kapteyn, J. Knobloch,G. Kurdi, N. Mahne, M. Manfredda, A. Martinelli, M. Murnane, E. Principi, L. Raimondi,S. Spampinati, C. Spezzani, M. Trovò, M. Zangrando, G. Chen, G. Monaco,K. A. Nelson, C. Masciovecchio,

Nanoscale transient gratings excited and probed by extreme ultraviolet femtosecond pulses. *Sci. Adv.* **5**, eaaw5805 (2019).

7. F. Bencivenga, F. Capotondi, L. Foglia, R. Mincigrucci, C. Masciovecchio, Extreme ultraviolet transient gratings. *Adv. Phys. X* **8**, 2220363 (2023).

8. A. A. Maznev, R. Mincigrucci, F. Bencivenga, V. Unikandanunni, F. Capotondi, G. Chen, Z. Ding, R. A. Duncan, L. Foglia, M. G. Izzo, C. Masciovecchio, A. Martinelli, G. Monaco, E. Pedersoli, S. Bonetti, K. A. Nelson, Generation and detection of 50 GHz surface acoustic waves by extreme ultraviolet pulses. *Appl. Phys. Lett.* **119**, 044102 (2021).

9. D. Naumenko, R. Mincigrucci, M. Altissimo, L. Foglia, A. Gessini, G. Kurdi, I. Nikolov, E. Pedersoli, E. Principi, A. Simoncig, M. Kiskinova, C. Massciovecchio, F. Capotondi, F. Bencivenga, Thermoelasticity of nanoscale silicon carbide membranes excited by extreme ultraviolet transient gratings: Implications for mechanical and thermal management. *ACS Appl. Nano Mater.* **2**, 5132–5139 (2019).

10. D. Ksenzov, A. A. Maznev, V. Unikandanunni, F. Bencivenga, F. Capotondi, A. Caretta, L. Foglia, M. Malvestuto, C. Masciovecchio, R. Mincigrucci, K. A. Nelson, M. Pancaldi, E. Pedersoli, L. Randolph, H. Rahmann, S. Urazhdin, S. Bonetti, C. Gutt, Nanoscale transient magnetization gratings created and probed by femtosecond extreme ultraviolet pulses. *Nano Lett.* **21**, 2905–2911 (2021).

11. K. Yao, F. Steinbach, M. Borchert, D. Schick, D. Engel, F. Bencivenga, R. Mincigrucci, L. Foglia, E. Pedersoli, D. D. Angelis, M. Pancaldi, B. Wehinger, F. Capotondi, C. Masciovecchio, S. Eisebitt, C. V. K. Schmising, All-optical switching on the nanometer scale excited and probed with femtosecond extreme ultraviolet pulses. *Nano Lett.* **22**, 4452–4458 (2022).

12. A. Kirilyuk, A. V. Kimel, T. Rasing, Ultrafast optical manipulation of magnetic order. *Rev. Mod. Phys*. **82**, 2731, 2784 (2010).

13. G. Cao, S. Jiang, J. Akerman, J. Weissenrieder, Femtosecond laser driven precessing magnetic gratings. *Nanoscale* **13**, 3746–3756 (2021).

14. A. V. Chumak, V. I. Vasyuchka, A. A. Serga, B. Hillebrands, Magnon spintronics. *Nat. Phys.* **11**, 453–461 (2015).

15. A. Barman, G. Gubbiotti, S. Ladak, A. O. Adeyeye, M. Krawczyk, J. Gräfe, C. Adelmann, S. Cotofana, A. Naeemi, V. I. Vasyuchka, B. Hillebrands, S. A. Nikitov, H. Yu, D. Grundler, A. V. Sadovnikov, A. A. Grachev, S. E. Sheshukova, J. Y. Duquesne, M. Marangolo, G. Csaba, W. Porod, V. E. Demidov, S. Urazhdin, S. O. Demokritov, E. Albisetti, D. Petti, R. Bertacco, H. Schultheiss, V. V. Kruglyak, V. D. Poimanov, S. Sahoo, J. Sinha, H. Yang, M. Münzenberg, T. Moriyama, S. Mizukami, P. Landeros, R. A. Gallardo, G. Carlotti, J. V. Kim, R. L. Stamps, R. E. Camley, B. Rana, Y. Otani, W. Yu, T Yu, G. E. W. Bauer, C. Back, G. S. Uhrig, O. V. Dobrovolskiy, B. Budinska, H. Qin, S. van Dijken, A. V. Chumak, A. Khitun, D. E. Nikonov, I. A. Young, B. W. Zingsem, M. Winklhofer, The 2021 Magnonics Roadmap. *J. Phys. Condens. Matter*. **33**, 413001 (2021).

16. J. E. Prieto, F. Heigl, O. Krupin, G. Kaindl, K. Starke, Prediction of huge x-ray faraday rotation at the GdN4,5threshold. *Phys. Rev. B* **66**, 172408 (2002).

17. K. Starke, F. Heigl, A. Vollmer, M. Weiss, G. Reichardt, G. Kaindl, X-ray magneto-optics in lanthanides. *Phys. Rev. Lett*. **86**, 3415–3418 (2001).

18. M. Hennecke, D. Schick, T. Sidiropoulos, F. Willems, A. Heilmann, M. Bock, L. Ehrentraut, D. Engel, P. Hessing, B. Pfau, M. Schmidbauer, A. Furchner, M. Schnuerer, C. von Korff Schmising, S. Eisebitt, Ultrafast element- and depth-resolved magnetization dynamics probed by

transverse magneto-optical Kerr effect spectroscopy in the soft x-ray range. *Phys. Rev. Res.* **4**, L022062 (2022).

19. I. Radu, C. Stamm, A. Eschenlohr, F. Radu, R. Abrudan, K. Vahaplar, T. Kachel, N. Pontius, R. Mitzner, K. Holldack, A. Föhlisch, T. A. Ostler, J. H. Mentink, R. F. L. Evans, R. W. Chantrell, A. Tsukamoto, A. Itoh, A. Kirilyuk, A. V. Kimel, T. Rasing, Ultrafast and distinct spin dynamics in magnetic alloys. *SPIN* **05**, 1550004 (2015).

20. M. van Kampen, C. Jozsa, J. T. Kohlhepp, P. LeClair, L. Lagae, W. J. M. de Jonge, B. Koopmans, All-optical probe of coherent spin waves. *Phys. Rev. Lett*. **88**, 227201 (2002).

21. T. G. Blank, S. Hermanussen, T. Lichtenberg, T. Rasing, A. Kirilyuk, B. Koopmans, A. V. Kimel, Laser-induced transient anisotropy and large amplitude magnetization dynamics in a Gd/FeCo multilayer. *Adv. Mater. Interfaces* **9**, 2201283 (2022).

22. A. Mekonnen, M. Cormier, A. V. Kimel, A. Kirilyuk, A. Hrabec, L. Ranno, T. Rasing, Femtosecond Laser Excitation of Spin Resonances in Amorphous Ferrimagnetic Gd1−xCox Alloys. *Phys. Rev. Lett.* **107**, 117202 (2011).

23. J. Van Kranendonk, J. H. Van Vleck, Spin waves. *Rev. Mod. Phys*. **30**, 1–23 (1958).

24. D. D. Stancil, A. Prabhakar, *Spin Waves Theory and Applications*. (Springer, 2009).

25. M. Binder, A. Weber, O. Mosendz, G. Woltersdorf, M. Izquierdo, I. Neudecker, J. R. Dahn, T. D. Hatchard, J. U. Thiele, C. H. Back, M. R. Scheinfein, Magnetization dynamics of the ferrimagnet CoGd near the compensation of magnetization and angular momentum. *Phys. Rev. B* **74**, 134404 (2006).

26. G. Shirane, V. J. Minkiewicz, R. Nathans, S. J. Pickart, H. A. Alperin, Spin-wave dispersion relation in Fe-Ni alloys. *J. Appl. Phys.* **39**, 383 (2008).

27. C. D. Stanciu, A. V. Kimel, F. Hansteen, A. Tsukamoto, A. Itoh, A. Kirilyuk, T. Rasing, Ultrafast spin dynamics across compensation points in ferrimagnetic GdFeCo: The role of angular momentum compensation. *Phys. Rev. B* **73**, 220402 (2006).

28. B. Pfau, S. Schaffert, L. Müller, C. Gutt, A. Al-Shemmary, F. Büttner, R. Delaunay, S. Düsterer, S. Flewett, R. Frömter, J. Geilhufe, E. Guehrs, C. M. Günther, R. Hawaldar, M. Hille, N. Jaouen, A. Kobs, K. Li, J. Mohanty, H. Redlin, W. F. Schlotter, D. Stickler, R. Treusch, B. Vodungbo, M. Kläui, H. P. Oepen, J. Lüning, G. Grübel, S. Eisebitt, Ultrafast optical demagnetization manipulates nanoscale spin structure in domain walls. *Nat. Commun.* **3**, 1100 (2012).

29. K. Kang, H. Omura, D. Yesudas, O. Lee, K.-J Lee, H.-W. Lee, T. Taniyama, G.-M. Choi, Spin current driven by ultrafast magnetization of FeRh. *Nat. Commun.* **14**, 3619 (2023).

30. C. Mathieu, C. Mathieu, J. Jorzick, A. Frank, S. O. Demokritov, A. N. Slavin, B. Hillebrands, B. Bartenlian, C. Chappert, D. Decanini, F. Rousseaux, E. Cambril, Lateral quantization of spin waves in micron size magnetic wires. *Phys. Rev. Lett.* **81**, 3968–3971 (1998).

31. C. Kim, S. Lee, H.-G. Kim, J.-H. Park, K.-W. Moon, J. Y. Park, J. M. Yuk, K.-J. Lee, B.-G. Park, S. K. Kim, K.-J. Kim, C. Hwang, Distinct handedness of spin wave across the compensation temperatures of ferrimagnets. *Nat. Mater.* **19**, 980–985 (2020).

32. C. W. Sandweg, M. B. Jungfleisch, V. I. Vasyuchka, A. A. Serga, P. Clausen, H. Schultheiss, B. Hillebrands, A. Kreisel, P. Kopietz, Wide-range wavevector selectivity of magnon gases in Brillouin light scattering spectroscopy. *Rev. Sci. Instrum.* **81**, 073902 (2010).

33. K. Jenni, S. Kunkemöller, A. Tewari, R. A. Ewings, Y. Sidis, A. Schneidewind, P. Steffens, A. A. Nugroho, M. Braden, Magnon dispersion in ferromagnetic SrRuO3. *Phys. Rev. B* **107**, 174429, (2023).

34. H. Man, Z. Shi, G. Xu, Y. Xu, X. Chen, S. Sullivan, J. Zhou, K. Xia, J. Shi, P. Dai. Direct observation of magnon-phonon coupling in yttrium iron garnet. *Phys. Rev. B* **96**, 100406 (2017).

35. L. Martinelli, D. Betto, K. Kummer, R. Arpaia, L. Braicovich, D. Di Castro, N. B. Brookes, M. M. Sala, G. Ghiringhelli. Fractional spin excitations in the infinite-layer cuprate CaCuO2. *Phys. Rev. X* **12**, 021041 (2022).

36. C. Liu, J. Chen, T. Liu, F. Heimbach, H. Yu, Y. Xiao, J. Hu, M. Liu, H. Chang, T. Stueckler, S. Tu, Y. Zhang, Y. Zhang, P. Gao, Z. Liao, D. Yu, K. Xia, N. Lei, W. Zhao, M. Wu. Long-distance propagation of short-wavelength spin waves. *Nat. Commun.* **9**, 738 (2018).

37. H. Yu, O. d'Allivy Kelly, V. Cros, R. Bernard, P. Bortolotti, A. Anane, F. Brandl, F. Heimbach, D. Grundler, Approaching soft x-ray wavelengths in nanomagnet-based microwave technology. *Nat. Commun.* **7**, 11255 (2016).

38. T. Hula, K. Schultheiss, F. J. T. Gonçalves, L. Körber, M. Bejarano, M. Copus, L. Flacke, L. Liensberger, A. Buzdakov, A. Kákay, M. Weiler, R. Camely, J. Fassbender, H. Schultheiss. Spin-wave frequency combs. *Appl. Phys. Lett.* **121**, 112404 (2022).

39. Z. Zhang, F. Y. Gao, J. B. Curtis, Z.-J. Liu, Y.-C. Chien, A. von Hoegen, M.T Wong, T. Kurihara, T. Suemoto, P. Narang, E. Baldini, K. A. Nelson. Three-wave mixing of anharmonically coupled magnons. arXiv:2301.12555 [cond-mat.mtrl-sci] (2023).

40. H. Schultheiss, K. Vogt, B. Hillebrands. Direct observation of nonlinear four-magnon scattering in spin-wave microconduits. *Phys. Rev. B* **86**, 054414 (2012).

41. L. Foglia, B. Wehinger, G. Perosa, R. Mincigrucci, E. Allaria, F. Armillotta, A. Brynes, R. Cucini, D. De Angelis, G. De Ninno, W. D. Engel, D. Fainozzi, L. Giannessi, N. N. Khatu, S. Laterza, E. Paltanin, J. S. Pelli-Cresi, G. Penco, D. Puntel, P. Rebernik Ribič, F. Sottocorona, M. Trovò, C. von Korff Schmising, K. Yao, C. Masciovecchio, S. Bonetti, F. Bencivenga. Nanoscale transient polarization gratings. arXiv:2310.15734 [cond-mat.mtrl-sci] (2023).
42. P. C. Johnsen, S. A. Ryan, C. Gentry, A. Grafov, H. Kapteyn, M. Murnane. A beamline for ultrafast extreme ultraviolet magneto-optical spectroscopy in reflection near the shot noise limit. *Rev. Sci. Instrum.* **94**, 033001 (2023).
43. C. S. Davies, J. Janušonis, A. V. Kimel, A. Kirilyuk, A. Tsukamoto, T. Rasing, R. I. Tobey. Towards massively parallelized all-optical magnetic recording. *J. Appl. Phys.* **123**, 213904 (2018).
44. V. Ukleev, M. Burian, S. Gliga, C. A. F. Vaz, B. Rösner, D. Fainozzi, G. Seniutinas, A. Kubec, R. Mankowsky, H. T. Lemke, E. R. Rosenberg, C. A. Ross, E. Müller, C. David, C. Svetina, U. Staub. Effect of intense x-ray free-electron laser transient gratings on the magnetic domain structure of Tm:YIG. *J. Appl. Phys.* **133**, 123902 (2023).
45. R. Mincigrucci, L. Foglia, D. Naumenko, E. Pedersoli, A. Simoncig, R. Cucini, A. Gessini, M. P. Kiskinova, G. Kurdi, N. Mahne, M. Manfredda, I. P. Nikolov, E. Principi, L. Raimondi, M. Zangrando, C. Massciovecchio, F. Capotondi, F. Bencivenga. Advances in instrumentation for FEL-based four-wave mixing experiments. *Nucl. Instrum. Methods Phys. Res., Sect. A* **907**, 132–148 (2018).
46. E. Allaria, D. Castronovo, P. Cinquegrana, P. Craievich, M. Dal Forno, M. B. Danailov, G. D'Auria, A. Demidovich, G. De Ninno, S. Di Mitri, B. Diviacco, W. M. Fawley, M. Ferianis, E. Ferrari, L. Froehlich, G. Gaio, D. Gauthier, L. Giannessi, R. Ivanov, B. Mahieu, N. Mahne, I.

Nikolov, F. Parmigiani, G. Penco, L. Raimondi, C. Scafuri, C. Serpico, P. Sigalotti, S. Spampinati, C. Spezzani, M. Svandrlik, C. Svetina, M. Trovo, M. Veronese, D. Zangrando, M. Zangrando. Two-stage seeded soft x-ray free-electron laser. *Nat. Photonics* **7**, 913–918 (2013).

47. B. L. Henke, E. M. Gullikson, J. C. Davis, X-ray interactions: Photoabsorption, scattering, transmission, and reflection at E = 50-30,000 eV, Z = 1-92. *At. Data Nucl. Data Tables* **54**, 181–342 (1993).

48. H. J. Eichler, P. Gunter, D. W. Pohl, *Laser-Induced Dynamic Gratings* (Springer-Verlag Berlin, 1986).
